# LC Resonant-Based Method for Permeability Interference Suppression in Magnetized Pipeline Eddy Current Testing

**DOI:** 10.3390/s26020680

**Published:** 2026-01-20

**Authors:** Lin Su, Yuxuan Li, Tong Cao, Shengping Li, Jie Zhang

**Affiliations:** 1 China Oil & Gas Pipeline Network Corporation Pipeline Technology Development Co., Ltd., Tianjin 300450, China; sulin@pipechina.com.cn (L.S.); liyuxuan@pipechina.com.cn (Y.L.); caotong@pipechina.com.cn (T.C.); 2 The School of Automation Engineering, University of Electronic Science and Technology of China, Chengdu 611731, China; zhj06_19@uestc.edu.cn

**Keywords:** LC resonant sensor, magnetized pipeline, eddy current testing (ECT), permeability interference suppression

## Abstract

In the eddy current testing (ECT) of magnetized ferromagnetic pipelines, permeability perturbations near defects cause magnetic distortion that primarily modulates the imaginary part of the ECT sensor’s impedance, leading to confusion between inner and outer wall defect signals. To address this interference, this study thoroughly analyzes the modulation mechanism of permeability changes on impedance and investigates the feasibility of detecting solely the real part to enhance discrimination reliability. This understanding leads to the proposal of a solution employing an LC resonant circuit, capitalizing on its characteristic of zero imaginary part impedance at the resonant frequency, to effectively suppress the permeability-related signal interference. Experimental results demonstrate the effectiveness of the proposed approach: the magnetization response test confirms the insensitivity of the LC sensor to permeability perturbations, and the defect discrimination experiment shows that the sensor achieves a standard deviation ratio of 2.25 and a peak-to-peak ratio of 4.42 between inner and outer wall defect signals. The findings indicate that the LC resonant sensor can reliably distinguish between inner and outer wall defects through simple amplitude thresholding, thereby improving the reliability of inspections for magnetized pipelines in industrial applications.

## 1. Introduction

Ferromagnetic pipelines are widely used in critical industries such as petroleum, natural gas, and nuclear energy due to their high strength and durability [1,2]. However, during long-term service, both the inner and outer walls of these pipelines are susceptible to defects such as corrosion and cracks, which may lead to catastrophic failures if not detected in a timely manner [3]. To ensure pipeline safety, nondestructive testing (NDT) methods are extensively employed for internal pipeline inspection. Among these, the combined detection mode of magnetic flux leakage (MFL) and eddy current testing (ECT) is widely adopted: MFL utilizes the leakage magnetic field signals at defects after magnetization to detect the presence and size of defects, while ECT employs the skin effect principle to distinguish whether defects are located on the inner or outer wall by analyzing the attenuation characteristics of eddy current signals [4,5,6,7,8].

However, conventional MFL methods often fail to achieve saturation magnetization due to the limited driving capability and design constraints of the in-pipe magnetizers [9]. Under these conditions, the presence of defects under magnetization severely distorts the magnetic field distribution within the pipe wall. Due to the nonlinear magnetization characteristics of ferromagnetic materials, defects directly induce strong spatial perturbations in magnetic permeability [10,11,12]. Specifically, outer wall defects compress magnetic flux lines around the defect, increasing magnetic field intensity in the inner wall region and reducing inner wall permeability. This permeability perturbation then affects the ECT sensor’s impedance signal via electromagnetic coupling—particularly the imaginary part (reactance), which is sensitive to permeability changes—leading ECT to misidentify outer wall defects as inner wall defects and impairing its discrimination capability.

To deal with this problem, various methods have been proposed to improve defect discrimination. For instance, Piao et al. [13] introduced a pulsed eddy current method using short pulses and differential coils to distinguish ID/OD defects via opposite signal polarities. Ou et al. [4,10] proposed a weak bias-magnetized dynamic permeability testing (DPT) method, where ID and OD defects exhibit distinct double-peak and single-peak waveforms, enabling clear differentiation. Ref. [14] explored pulsed eddy current techniques that employ the richness of time-domain signals for defect characterization. Chae et al. [1] discriminates between internal and external defects by leveraging phase differences in signals, optimized through DC magnetization and frequency parameters for enhanced sensitivity. However, these methods are prone to being affected by motion-induced artifacts, such as lift-off variations or attitude perturbations during inspection, which distort signal waveforms and obscure characteristic features, thereby limiting reliable pattern recognition.

To address the interference from permeability-induced signals in magnetization conditions, effectively suppressing the imaginary component of the impedance is crucial. Among various ECT circuit configurations, the LC resonant circuit offers a targeted solution. When operating at its resonant frequency, the LC circuit exhibits a unique characteristic where the imaginary part of the impedance approaches zero, thereby isolating the real component of the response. This real-part isolation effectively separates conductivity-related signals from permeability-induced interference, directly addressing the challenge of permeability perturbation in weakly magnetized pipelines.

LC resonant circuits have found extensive applications in electromagnetic sensing and nondestructive testing. For example, Wu et al. [15] developed a self-resonance ECT system for carbon fiber reinforced polymers (CFRPs), using LC resonance at 500 kHz to enhance defect detection sensitivity with an improved signal-to-noise ratio. In metal debris monitoring, Shi et al. [16] designed a microsensor based on resistance–inductance parameters, enabling high-sensitivity detection of ferromagnetic and non-ferromagnetic particles in oil. However, most existing research focuses on non-magnetic or weakly magnetic materials [17], with limited analysis of resonant behavior in ferromagnetic materials under magnetization—particularly regarding real–imaginary component isolation during permeability perturbations.

This paper provides a theoretical analysis and experimental validation to address a critical challenge in eddy current testing under weak magnetization: distinguishing between inner and outer wall defects with permeability interference. We first elucidate the mechanism by which magnetic permeability perturbations predominantly affect the imaginary component of the impedance signal. Based on this theoretical insight, we demonstrate that the established technique of LC resonance offers a targeted solution. By operating a sensor at its resonant frequency, the imaginary part is nullified, effectively isolating the conductivity-sensitive real-part response. Comparative experiments on magnetized steel plates with artificial defects confirm that this application of LC resonance provides superior clarity for defect location discrimination compared to conventional differential sensors. This study is built upon the theoretical framework of magnetic permeability perturbation [5] and boundary magnetic perturbation [10]. By rigorously analyzing the underlying mechanism and validating the efficacy of an LC-resonance-based approach, this work offers a practical and well-founded solution for enhancing defect discrimination in industrial applications.

## 2. Method

### 2.1. Differences in Real and Imaginary Part Responses to Magnetic Permeability in ECT

In a uniform and isotropic ferromagnetic material, the normalized impedance change of an eddy current testing (ECT) coil is related to the surface impedance Z, given by:

(1) $$\begin{array}{c} Z=R+jX=\frac{1}{\sigma \delta }+j\frac{1}{\sigma \delta }=\frac{1+j}{\sigma \delta } \end{array}$$

where $\delta =\sqrt{\frac{2}{\omega \mu \sigma }}$ is the skin depth (m), ω is the angular frequency (rad/s), μ is the magnetic permeability (H/m), and σ is the conductivity (S/m). The real part (*R*) and the imaginary part (*X*) are equal, resulting in a phase angle $\phi =arctan\left(X/R\right)=45^{\circ }$. When the material undergoes uniform changes, uniform variations in conductivity and permeability lead to proportional changes in *R* and *X* (ΔR=ΔX), while the phase angle ϕ remains unchanged.

However, the presence of defects fundamentally disrupts this uniformity. An inner wall defect represents a localized abrupt change in both electrical conductivity Δσ and magnetic permeability (Δμ). It therefore directly disturbs the eddy current field, inducing significant and correlated variations in both the loss component R and the reactive component X. This mechanism forms the basis of conventional ECT.

In contrast, an outer wall defect influences the signal primarily through magnetic field distortion. Even when the defect depth exceeds the standard skin depth—eddy currents do not directly interact with the defect—the defect still locally perturbs the magnetic permeability (Δμ) under weak magnetization. This permeability variation modulates the magnetic field distribution within the pipe wall and predominantly affects the imaginary part X of the coil impedance, introducing interference that is difficult to distinguish from the response of a real inner wall defect.

**Figure 1:**
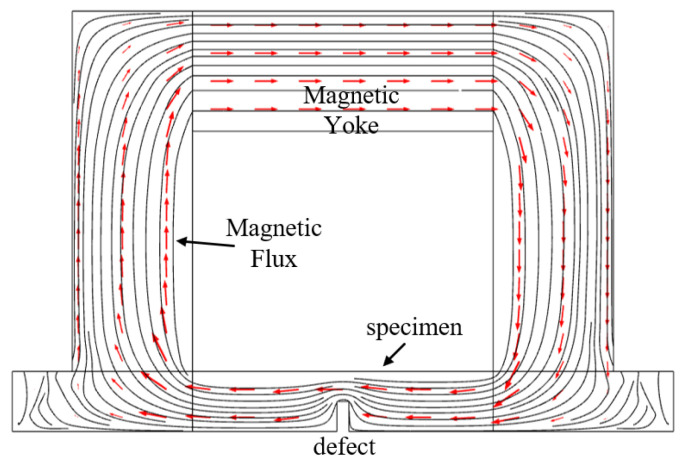
Magnetic flux distribution in the defective area under magnetization.

To quantitatively illustrate the phenomenon of magnetic permeability perturbation induced by defects under magnetization, a two-dimensional magneto-static simulation was performed using COMSOL Multiphysics 5.6. The model geometry, shown in Figure 1, consisted of a ferromagnetic specimen made of low-carbon steel, representing a pipe wall. The material was defined with nonlinear magnetic properties according to its actual B-H curve, including an initial relative permeability of 4000 and an electrical conductivity of 10 MS/m. A rectangular outer wall defect was introduced into the specimen, which was surrounded by a sufficiently large air domain to approximate far-field boundary conditions. A uniform static magnetic field with a strength of H = 1,000,000 A/m was applied to magnetize the specimen, implemented as a boundary condition to simulate a well-defined magnetized state. The simulation employed the “Magnetic Fields, No Currents” physics interface, which solves the governing equation for the magnetic scalar potential. The outer boundaries of the air domain were set to magnetic insulation to confine the magnetic field within the computational domain. A fine mesh was applied around the defect region to ensure accurate resolution of the magnetic flux and permeability distributions.

The simulated magnetic flux lines, represented by black streamlines, demonstrate significant distortion and concentration around the outer wall defect. A notable magnetic flux crowding effect occurs at the inner wall region directly opposite the defect, indicating localized field intensification. This effect stems from the disruption of the magnetic circuit by the defect, causing flux to be redirected and concentrated along lower reluctance paths, particularly at the inner wall opposite the defect.

**Figure 2:**
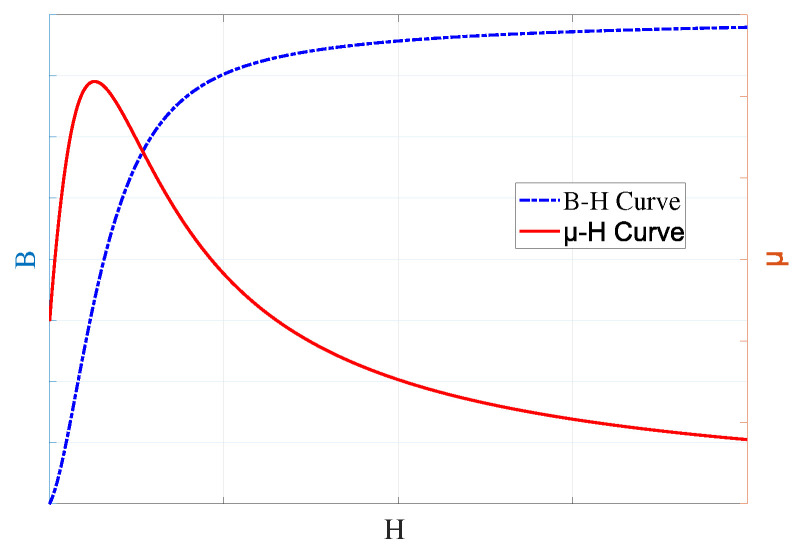
Nonlinear B-H curve and corresponding Permeability (μ) of a ferromagnetic material.

The distortion of magnetic flux distribution directly induces significant changes in material permeability, a consequence of the intrinsic nonlinear magnetic properties of ferromagnetic materials. As illustrated by the B-H curve and the permeability curve (μ−H) in Figure 2, the permeability of ferromagnetic materials is not a constant but exhibits a strong nonlinear dependence on the magnetic field strength (H). The μ−H curve clearly shows that the permeability initially increases with the magnetic field, reaches a maximum, and then decreases. This nonlinear relationship implies that any spatial variation in the magnetic field, such as that caused by a defect, will inevitably induce a corresponding spatial perturbation in the permeability (μ).

**Figure 3:**
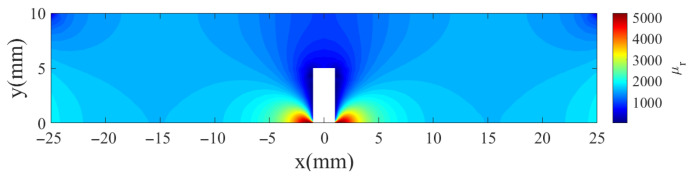
The $\mu_{r}$ perturbation under magnetization.

The direct interplay between the distorted magnetic field and the material’s nonlinear μ−H characteristic produces a highly non-uniform permeability perturbation, as illustrated by the simulated permeability distribution under magnetized conditions in Figure 3. The results reveal a distinct spatial pattern: the edges of the outer wall defect, where the local magnetic field strength (H) is relatively weak, exhibit a substantial increase in permeability; conversely, the area on the inner wall directly opposite the defect, where the magnetic flux crowding effect creates a locally strong H-field, shows a marked decrease. Thus, the simulated μ distribution confirms that an outer wall defect under magnetization creates a strong, spatially varying permeability perturbation in the inner wall ($\nabla \left(\Delta \mu \right)$ is large).

This perturbation affects the different components of the coil impedance in distinct ways. The inductive reactance X is proportional to the magnetic energy stored in the system ($W_{m}\propto \int \mu |H{|}^{2}dV$), and its variation $\Delta X_{\mu }$ primarily reflects the spatially averaged effect of the permeability perturbation, ⟨$\langle \Delta \mu \rangle$⟩. For small perturbations in permeability Δμ, the change in reactance can be approximated as:

(2) $$\begin{array}{c} \Delta X_{\mu }\approx \frac{\partial X}{\partial \mu }\Delta \mu \propto \int_{V}{\left|H\right|}^{2}\Delta \mu dV \end{array}$$

When the permeability perturbation is spatially distributed, the integral simplifies to an averaged effect $\langle \Delta \mu \rangle$. Therefore, the global response can lead to false signals even without genuine defects.

The real part $R_{\mu }$ corresponds to the eddy current loss ($P_{loss}\propto \int |J{|}^{2}dV$). Non-uniform Δμ distorts the eddy current paths, diluting the effective current density, and thus suppressing the increase in loss. Consequently, ΔR is not only related to ⟨Δμ⟩ but is also suppressed by the permeability gradient ∇(Δμ).

(3) $$\begin{array}{c} \Delta R_{\mu }\propto \frac{\langle \Delta \mu \rangle }{1+\langle {\left(\nabla (\Delta \mu )\right)}^{2}\rangle /\left(j\omega \mu \sigma \right)} \end{array}$$

reflects that the increase in loss due to the average permeability rise $\langle \Delta \mu \rangle$ is suppressed when the squared gradient term $\langle {\left(\nabla \left(\Delta \mu \right)\right)}^{2}\rangle$ s large. The denominator thus incorporates a damping factor related to the non-uniformity of the permeability field.

Under magnetization and in the presence of an outer-wall defect, the permeability perturbation is highly non-uniform. As shown in Figure 3, Δμ exhibits strong spatial gradients near the defect edges and the opposite inner-wall region. In such cases, the gradient term dominates the denominator of Equation (3):

(4) $$\begin{array}{c} \langle {\left(\nabla \left(\Delta \mu \right)\right)}^{2}\rangle \gg j\omega \mu \sigma \cdot \langle \Delta \mu \rangle \end{array}$$

Consequently, the magnitude of $\Delta R_{\mu }$ becomes small compared to the unperturbed case. In contrast, the imaginary part change $\Delta X_{\mu }\propto \langle \Delta \mu \rangle$ remains directly proportional to the average permeability variation. This leads directly to the key inequality:

(5) $$\begin{array}{c} \left|\Delta X_{\mu }\right|\gg \left|\Delta R_{\mu }\right| \end{array}$$

It indicates that the permeability perturbation is manifested almost entirely in the signal from the imaginary part ($X_{\mu }$), while its impact on the signal from the real part ($R_{\mu }$) is negligible. Consequently, traditional detection methods relying on the imaginary part/phase signal are prone to misinterpreting the permeability interference caused by outer wall defects as signals from inner wall defects.

Based on the distinctions described above, this study proposes a core thesis: interference suppression can be achieved if a physical quantity that is sensitive only to genuine defects, yet insensitive to permeability perturbations, can be selectively measured. The real part of impedance (R) represents precisely such an ideal quantity.

### 2.2. Discrimination Capability of the Real Part Signal Between Inner and Outer Wall Defects

After demonstrating that the real-part signal can more effectively suppress magnetic permeability interference compared to the imaginary-part signal, a key question arises: Can the real-part signal still generate sufficiently large and distinguishable responses to genuine inner and outer wall defects?

For an inner wall defect, which constitutes a localized abrupt change in both electrical conductivity (σ) and magnetic permeability (μ), the resultant change in the real part of the impedance can be expressed as:

(6) $$\Delta R_{ID}=\left(\frac{\partial R}{\partial \sigma }\right)\Delta \sigma +\left(\frac{\partial R}{\partial \mu }\right)\Delta \mu_{ID}=R\left(-\frac{\Delta \sigma }{2\sigma }+\frac{\Delta \mu_{ID}}{2\mu }\right)$$

While for an outer wall defect, whose influence is primarily manifested as an indirect magnetic permeability perturbation (Δμ) under weak magnetization, while the conductivity remains largely unchanged, the resultant change in the real part is given by:

(7) $$\Delta R_{OD}=\left(\frac{\partial R}{\partial \mu }\right)\Delta \mu_{OD}=\frac{R\Delta \mu_{OD}}{2\mu }$$

Comparing the signal strength between them:

(8) $$\left|\frac{\Delta R_{ID}}{\Delta R_{OD}}\right|=\left|\frac{-\frac{R}{2\sigma }\Delta \sigma +\frac{R}{2\mu }\Delta \mu_{ID}}{\frac{R}{2\mu }\Delta \mu_{OD}}\right|\approx \left|-\frac{\mu }{\sigma }\cdot \frac{\Delta \sigma }{\Delta \mu_{OD}}+\frac{\Delta \mu_{ID}}{\Delta \mu_{OD}}\right|$$

To quantitatively assess the defect discrimination capability, an estimation was conducted based on the specific parameters derived from the above simulation. In the simulated model, the intact magnetized pipeline material was characterized by a relative permeability of $\mu_{r}$ = 1622 and an electrical conductivity of $\sigma =10^{7}$ S/m. For an inner wall defect, whose depth significantly exceeds the standard skin depth, the defect region can be approximated as a loss of material, effectively transitioning to air like electromagnetic properties. Consequently, the corresponding variations are defined as a complete change in properties: the conductivity variation Δσ is set to $-10^{7}$ S/m, and the relative permeability variation $\Delta \mu_{rID}$ is set to −1622. In contrast, an outer wall defect primarily induces a magnetic permeability perturbation due to factors like stress concentration under magnetization. Based on the simulation results, the relative permeability change caused by an outer wall defect, $\Delta \mu_{rID}$ is −594. Substituting these parameters into the derived equation yields the following calculation:

(9) $$\left|\frac{\Delta R_{ID}}{\Delta R_{OD}}\right|=5.46$$

This calculation result indicates that, under this simulation setup, the real-part signal response induced by an inner wall defect is approximately 5.46 times stronger than that caused by an outer wall defect, underscores the potential effectiveness of a detection strategy focused on the real part of the impedance for reliably discriminating between defect locations.

### 2.3. LC Resonant Eddy Current Sensor

**Figure 4:**
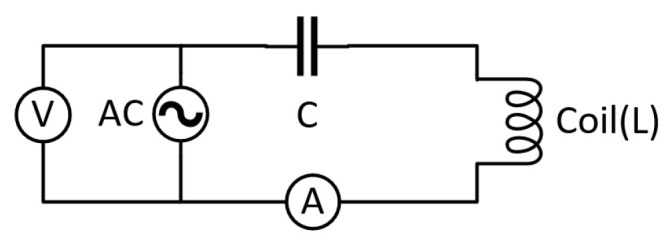
Schematic of LC Resonant Eddy Current Circuit.

To leverage the real part response, we employ an LC resonant sensor (as shown in Figure 4). The fundamental principle can be understood through both circuit theory and the sensor’s interaction with materials.

For a series LC circuit, the impedance is given by:

(10) $$Z_{LC}=R+j\left(\omega L-\frac{1}{\omega C}\right)$$

where L is the coil inductance, C is the tuning capacitance, and R is the series resistance of the circuit. At the resonant frequency $\omega_{r}=1/LC$, the inductive and capacitive reactances cancel exactly:

(11) $$\omega_{r}L=\frac{1}{\omega_{r}C}\Rightarrow X=0$$

This cancellation yields a purely resistive impedance $Z_{LC}=R$. The phenomenological significance is crucial: at resonance, the circuit becomes insensitive to any parameter that primarily affects the reactive component, including magnetic permeability variations.

When the sensor interacts with a conductive material, the impedance changes can be modeled as:

(12) $$Z=\left(R_{0}+\Delta R\right)+j\left[\omega \left(L_{0}+\Delta L\right)-\frac{1}{\omega C}\right]$$

where $R_{0}$ and $L_{0}$ represent the intrinsic resistance and inductance of the sensor coil in air, where ΔR is dominated by conductivity changes (eddy current losses) and ΔL is primarily influenced by permeability variations. At resonance, this simplifies to:

(13) $$Z_{LC}=R_{0}+\Delta R+j\omega_{r}\Delta L$$

The key innovation lies in the measurement strategy: instead of measuring the complex impedance (both real and imaginary parts), we measure only the magnitude $\left|Z_{LC}\right|=\sqrt{{\left(R_{0}+\Delta R\right)}^{2}+{\left(\omega_{r}\Delta L\right)}^{2}}$. When the circuit is precisely tuned to resonance with high quality factor $\omega_{r}L/R\gg 1$, the condition $R_{0}\gg \omega_{r}\Delta L$ ensures that:

(14) $$\left|Z\right|\approx R_{0}+\Delta R+𝒪\left(\frac{{\left(\omega_{r}\Delta L\right)}^{2}}{2R_{0}}\right)\approx R_{0}+\Delta R$$

This demonstrates that the permeability-dependent term ΔL contributes only a negligible second-order correction to the measured magnitude.

This analytical model confirms that resonance operation combined with magnitude detection provides a natural filtering mechanism: it preserves sensitivity to conductivity changes (genuine defects) while rejecting first-order interference from permeability perturbations.

## 3. Experimental Setup

**Figure 5:**
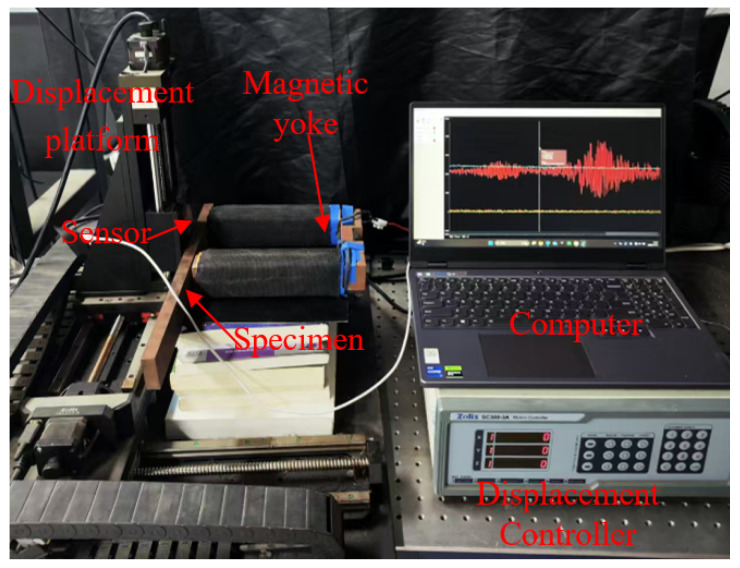
Schematic of the experimental platform.

**Figure 6:**
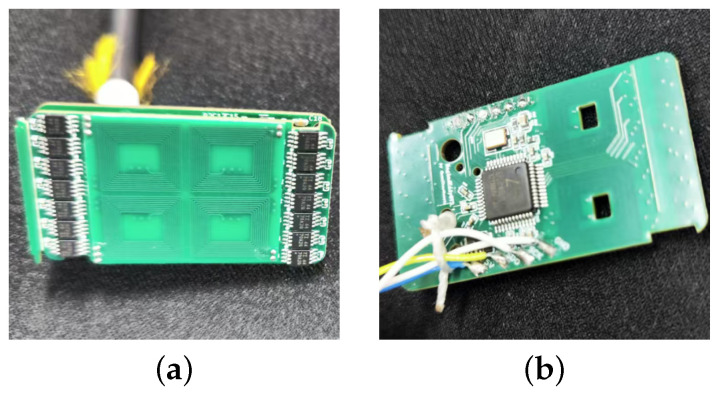
Sensors for the experiment. (**a**) Differential sensor. (**b**) LC resonant sensor.

The experimental setup used to validate the proposed LC resonant eddy current testing method under weak magnetization is shown in Figure 5. The system includes a computer-controlled linear displacement platform, an excitation/detection unit, and a magnetized specimen. Both the conventional differential sensor (Figure 6a) and the proposed LC resonant sensor (Figure 6b) are mounted on the platform. They were operated under identical conditions to ensure a fair comparison: scanning at a constant speed of 1 mm/s to minimize motion-induced interference, with an excitation frequency of 8 MHz, and a fixed lift-off of 2 mm. At this frequency, the calculated skin depth is approximately 0.056 mm. This shallow penetration confines the eddy currents to a thin surface layer, ensuring that the response is primarily sensitive to inner wall defects, while signals from deeper outer wall defects are attenuated.

The differential eddy current sensor comprises an excitation coil and a differential detection array. The excitation coil has 126 turns, with an inner rectangular dimension of 5.7 mm × 15.5 mm and an outer dimension of 26.5 mm × 16.5 mm. The detection system consists of two pairs of differential coils (four coils total), each with 60 turns, an inner dimension of 4.5 mm × 6.4 mm, and an outer dimension of 10 mm × 11.4 mm. This configuration is high sensitivity to impedance changes in both real and imaginary parts. The LC resonant sensor features a single circular coil with 16 turns, an inner diameter of 5 mm, and an outer diameter of 10 mm. It was designed as a series resonant circuit operating at 8 MHz. The circuit consisted of a capacitor with a value of C = 56 pF and a custom-wound coil with an inductance of L ≈ 7.07 μH. At resonance, the impedance of the circuit is minimized and purely resistive, effectively isolating the resistive component (related to eddy current losses) from the reactive component.

The magnetization system employs a U-shaped yoke made of a pure iron core (industrial pure iron DT4C), with 200-turn excitation coils wound on each of the two magnetic poles. The excitation current I was controlled by a programmable DC power supply and continuously adjusted in the range of 0 to 5 A to generate a magnetic field. At the maximum current of 5 A, the magnetic field strength within the specimen was calculated be approximately 10 kA/m. This condition is defined as ‘magnetization but not saturation’, as it is significantly below the saturation level of the material but sufficient to induce measurable magnetic permeability perturbations. Four test specimens were used, each with a width of 30 mm and a length of 200 mm. These include:

A calibration specimen set: a 1 mm thick aluminum plate (grade 6061-T6) without defects and a 1 mm thick iron plate (grade DT4C) without defects, used to independently verify the differential effects of the magnetic field on non-ferromagnetic (aluminum) and ferromagnetic (iron) materials.

**Figure 7:**
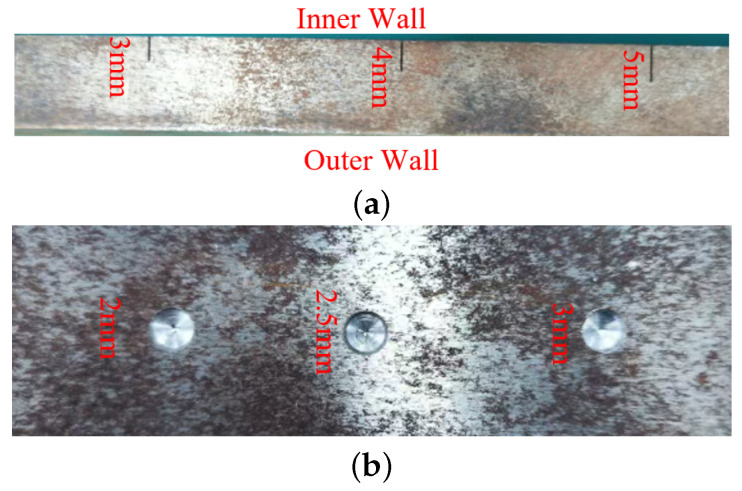
Schematic of the specimen and defects. (**a**) Cracks. (**b**) pits.

To verify the capability of different methods in internal and external wall defects discrimination, two defective specimens were designed. The first specimen, as shown in Figure 7a, is made of iron with a thickness of 15 mm. Rectangular notches with a width of 0.5 mm and depths of 3 mm, 4 mm, and 5 mm were machined to simulate inner and outer wall cracks. The second specimen (Figure 7b) was fabricated from the same base material but with a reduced thickness of 10 mm. It contains three circular pits, each with a diameter of 6 mm and depths of 2 mm, 2.5 mm, and 3 mm, respectively. These artificial defects were introduced to investigate the effect of different flaw geometries on the structural response. Signals obtained by scanning the surface containing the defects (simulating the inner wall of the pipe) were labeled as “inner wall defect” data, while signals from the opposite, defect-free side were labeled as “outer wall defect” data, thereby simulating the inspection of defects from both the internal and external surfaces of a pipe.

## 4. Result and Discussion

### 4.1. Permeability Response Test

To verify the difference in sensitivity to magnetic permeability perturbations between the LC resonant sensor and the differential sensor, an experiment was conducted under controlled dynamic magnetization conditions to compare the signal responses of the two sensors to different material specimens. A periodically varying dynamic magnetic field was generated by an electromagnetically controlled yoke, which was driven by a programmable DC power supply that output a triangular wave excitation current sweeping linearly from 0 A to 5 A. Under these conditions, the LC resonant sensor and the differential sensor were used to acquire signals from the surface of iron (ferromagnetic) and aluminum alloy (non ferromagnetic). By directly comparing the signal differences, the anti interference performance of the sensors against permeability perturbations could be effectively evaluated.

**Figure 8:**
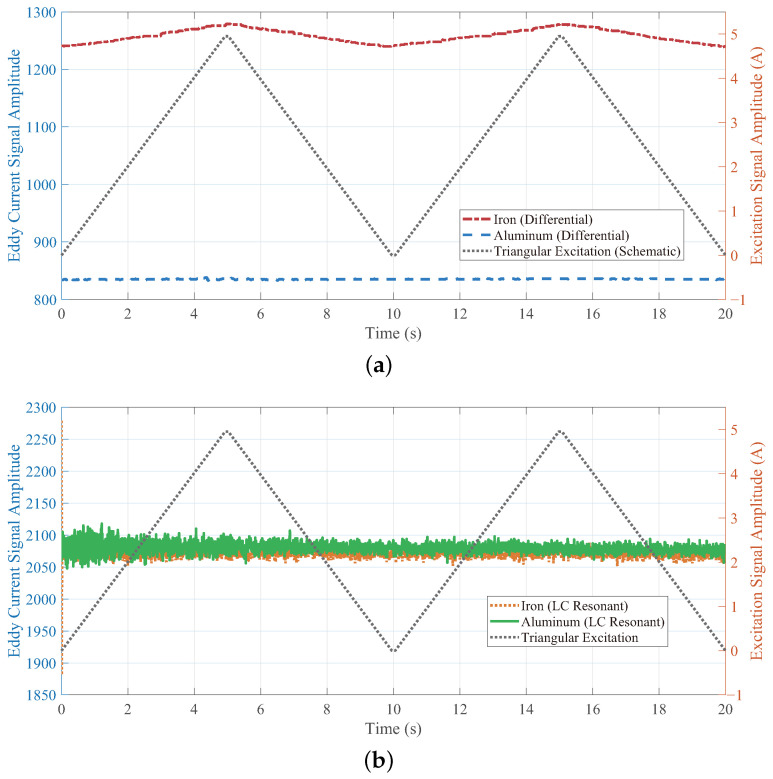
Comparison of eddy current responses under triangular magnetic field excitation. (**a**) Differential sensor signal. (**b**) LC resonant sensor signal.

As shown in Figure 8a, the signals from the differential sensor exhibit a clear distinction between the ferromagnetic (iron) and non-ferromagnetic (aluminum alloy) materials, which is dominated by magnetic permeability. Although iron and aluminum alloy have comparable electrical conductivities, the baseline signal amplitude from the iron specimen is significantly higher than that from aluminum. This indicates that, for the differential sensor, permeability has a pronounced influence on the signal in ferromagnetic materials. Furthermore, the iron signal shows evident modulation synchronized with the excitation current, resulting from magnetic-field-induced changes in relative permeability. The aluminum signal remains stable because its permeability is unaffected by the field. This contrast confirms that the magnetic field affects the signal only indirectly through permeability perturbations in ferromagnetic materials.

In contrast, Figure 8b presents the results for the LC resonant sensor. The LC sensor displays negligible nonlinearity, with consistent signal levels throughout the current range. The signal intensities for iron and aluminum are comparable and remain constant as the magnetic field varies, demonstrating that the LC sensor is largely insensitive to permeability perturbations. This stability aligns with its design principle: at resonance, the sensor measures only the real part of the impedance, which is insensitive to permeability variations, as established in Equation (5).

### 4.2. Performance Comparison of Differential and LC Resonant Sensors in Defects Detection

To accurately evaluate the capability of both sensors in discriminating between inner and outer wall defects under magnetized conditions, a fixed direct current of 5 A was applied to the electromagnetic yoke. The sensors were then scanned over the surface of the defective specimens (Figure 7) at a constant speed of 1 mm/s to acquire signal data from the defect regions.

**Figure 9:**
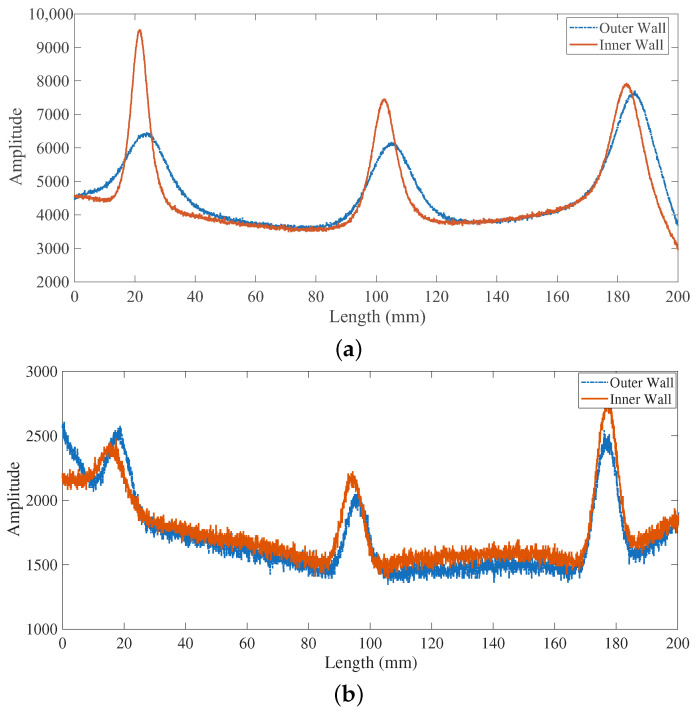
The comparison between inner wall defects and outer wall cracks signal. (**a**) cracks signal. (**b**) pits signal.

Figure 9 presents the signals obtained from the differential eddy current sensor when scanning over inner and outer wall defects. As anticipated by the theoretical analysis in Section 2.1, the sensor’s response, which is sensitive to both the real and imaginary components of the impedance, exhibits significant and morphologically similar signals on both sides. Such a condition makes it challenging to distinguish whether a signal originates from an inner wall defect (characterized by both Δσ and Δμ) or a false indication caused by the permeability perturbation of an outer wall defect (primarily Δμ). In a practical inspection environment, this ambiguity would be exacerbated by factors such as sensor lift-off variation and motion-induced noise, further crippling the reliability of defect discrimination.

**Figure 10:**
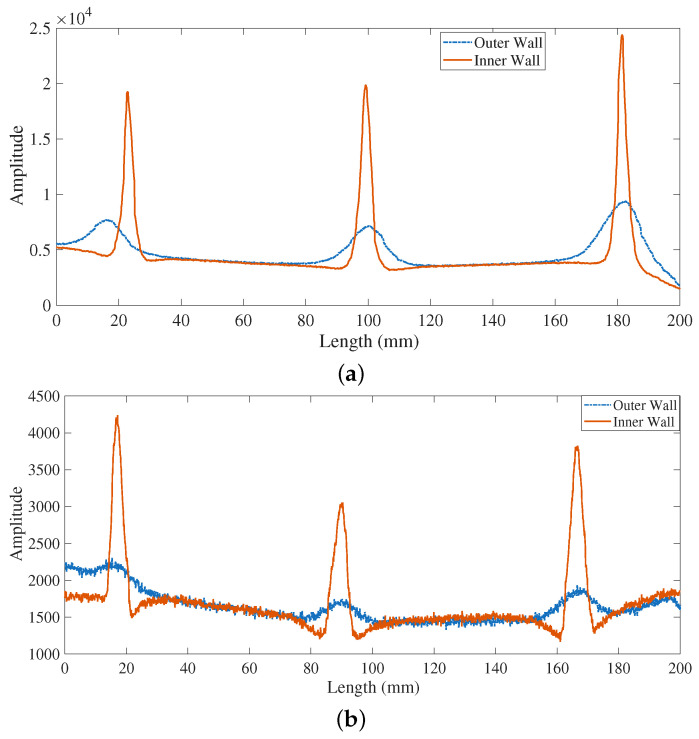
The comparison between inner wall defects and outer wall pits signal. (**a**) cracks signal. (**b**) pits signal.

In contrast, the performance of the proposed LC resonant sensor (as depicted in Figure 10) aligns well with the intended mechanism. The sensor produces a pronounced and clear signal when scanning across an inner wall defect. Conversely, its response to an outer wall defect is significantly attenuated, exhibiting a severely diminished amplitude. As established in Equation (5), magnetic permeability perturbations caused by an outer wall defect exert little influence on the real part R. Meanwhile, an inner wall defect—involving an abrupt conductivity change—induces a substantial variation in eddy current distribution, leading to a strong response in R, as captured by Equation (6). The significant amplitude disparity between inner and outer wall defects offers a simple and robust discrimination criterion: signals exceeding a predetermined threshold can be confidently classified as inner wall defects.

To quantify the amplitude variation of signals induced by defects, the peak-to-peak value of the sensor output was adopted as a direct metric. The comparative results are detailed in Table 1, revealing a fundamental disparity in the signal characteristics generated by the two sensors.

The LC resonant sensor demonstrates exceptional amplitude discrimination. For crack-type defects, the peak-to-peak value for an inner wall defect (16,739) is approximately 3.29 times greater than that for an outer wall defect (5090). This contrast is even more pronounced for pit-type defects, with a ratio of approximately 4.42 (2645 vs. 598). This significant amplification of inner wall defect signals, coupled with strong attenuation of outer wall signals, creates a clear amplitude-based criterion for defect localization.

Conversely, the differential sensor exhibits a much weaker amplitude contrast between defect types. The signal peak-to-peak ratio between inner and outer wall defects is only about 1.29 for cracks (3389 vs. 2620) and approximately 1.18 for pits (1034 vs. 872). Since the signals from both types of defects fall within a comparable amplitude range, distinguishing their locations based solely on magnitude becomes fundamentally challenging. This quantitative result aligns with and explains the qualitative observation of similar signal morphology from both defect types shown in Figure 9a, highlighting a key limitation of the conventional differential approach.

The standard deviation further reveals the dispersion characteristics of the signal responses. As shown in Table 2, for both types of defects, the LC sensor exhibits a notable difference in the standard deviation between inner and outer wall signals. Specifically, the ratio of the standard deviations for crack signals (inner to outer) is approximately 2.25 (3322/1477), and 2.17 (476/219) for pit signals, both exceeding 2. In contrast, the corresponding ratios for the differential sensor are much lower—approximately 1.25 (1273/1019) for cracks and even less than 1 (about 0.88, 269/306) for pits. Clearly, the inner-to-outer wall standard deviation ratios for the LC sensor are significantly higher than those for the differential sensor. This is because the LC sensor produces a strong and consistent response to conductivity variations caused by inner wall defects, while effectively suppressing interference from permeability perturbations primarily associated with outer wall defects. This results in a distinct contrast: high dispersion for inner wall signals and low dispersion for outer wall signals. In comparison, the differential sensor responds similarly to both types of defects, leading to significant overlap in signal amplitude distributions and minimal differences in dispersion.

In summary, this study quantitatively demonstrates via both signal amplitude and statistical distribution that the LC sensor, due to its physical mechanism of sensitivity to conductivity changes and suppression of permeability perturbations, can establish a clearer boundary between inner and outer wall defect signals. This provides a superior detection foundation for reliable defect localization.

It should be emphasized that although the two sensors differ in structure and signal processing, these differences are a necessary result of implementing their respective core measurement principles. The differential sensor is inherently sensitive to both the real and imaginary parts of impedance, making it susceptible to interference from permeability perturbations. In contrast, the proposed single-coil LC resonant configuration is the most direct way to operate in a resonant state, thereby suppressing the imaginary component. Experimental results confirm that its core advantage—selective suppression of outer wall defect signals—is a direct manifestation of this principle. Permeability response tests (Figure 8) indicate that the LC sensor is inherently less sensitive to permeability changes, while defect identification tests verify its selective response to genuine conductivity variations. Therefore, the performance improvement observed in this study fundamentally stems from the physical quantity it measures (the real part).

It is worth noting that while this study validates the principle under controlled conditions, practical application of the LC resonant method still faces the challenge of resonant frequency stability. Factors such as temperature variations, dynamic lift-off, and electromagnetic coupling with the specimen may cause detuning, potentially affecting its ability to suppress permeability interference. This study mitigated these effects through the use of stable components and pre-calibration to complete the proof of principle. To ensure robust performance under real-world operating conditions in the future, further work will focus on developing active frequency tracking mechanisms (such as phase-locked loops) or advanced signal processing schemes capable of compensating for detuning in real time.

## 5. Conclusions

This study addresses the critical challenge of permeability interference in eddy current testing for magnetized pipelines, where signal distortion impedes reliable defect discrimination. A thorough theoretical analysis revealed that magnetic permeability perturbations, primarily induced by outer wall defects under non-saturated magnetization, dominantly modulate the imaginary part of the sensor impedance. Based on this insight, an LC resonant eddy current sensor was applied, capitalizing on its operation at resonance where the impedance is purely real (Z|≈R), to effectively suppress this interference. Experimental results confirmed the sensor’s insensitivity to permeability perturbations and its superior performance in discriminating inner wall from outer wall defects, achieving a significant standard deviation ratio of 2.25 and a peak-to-peak signal ratio of 4.42. The key advantage of this method is the enablement of simple, reliable amplitude-based thresholding for defect classification, which significantly enhances inspection reliability under industrially relevant magnetization conditions.

While the COMSOL simulations and experimental validation in this study were performed on a specific low-carbon steel with rectangular notches under a defined non-saturated magnetization condition, the underlying physical mechanism of permeability perturbation is general to ferromagnetic materials. The key conclusion—that permeability interference dominantly affects the imaginary part of the impedance and can be effectively suppressed by an LC resonant sensor detecting the real part—is therefore widely applicable. However, it is important to note that the quantitative performance metrics may vary with material magnetic properties (e.g., B-H curve), defect geometry (affecting the spatial gradient of permeability perturbation), and the exact level of magnetization. Furthermore, it is crucial to acknowledge that realistic inspection conditions—characterized by complex defect morphology, environmental noise, lift-off variations, and changing material properties—will necessitate more sophisticated signal processing approaches. Future work will systematically study key parameters (material, defect geometry, magnetization) to define operational boundaries for industrial use. The ultimate goal is to validate the method through extensive field trials, developing it into a reliable system for practical pipeline inspection.

## Figures and Tables

**Table 1:** Peak-to-peak value comparison between inner wall defects signal and outer wall defects signal.

Walls	LC Sensor	Differential Sensor
Outer (crack|pit)	5090|598	2620|872
Inner (crack|pit)	16,739|2645	3389|1034

**Table 2:** Standard Deviation comparison between inner wall defect signals and outer wall defect signals.

Walls	LC Sensor	Differential Sensor
Outer (crack|pit)	1477|219	1019|306
Inner (crack|pit)	3322|476	1273|269

## Data Availability

The raw data supporting the conclusions of this article will be made available by the authors on request.
